# ACCEPTABILITY AMONG DEMENTIA CAREGIVERS OF THE ONLINE SELF-GUIDED ACT FOR CAREGIVERS PROGRAM

**DOI:** 10.1093/geroni/igae098.4106

**Published:** 2024-12-31

**Authors:** Gavin Green, Josey Batura, Heather Kelley, Jacob Gossner, Ty Aller, Michael Levin, Elizabeth Fauth

**Affiliations:** Utah State University, Logan, Utah, United States; Utah State University, Logan, Utah, United States; Utah State University, Logan, Utah, United States; Utah State University, Logan, Utah, United States; Utah State University, Logan, Utah, United States; Utah State University, Logan, Utah, United States; Utah State University, Logan, Utah, United States

## Abstract

Acceptance and Commitment Therapy (ACT) is an effective therapeutic intervention for stress and mental health concerns across various populations. Our study evaluates the acceptability of an online self-guided ACT program tailored specifically for dementia caregivers via a longitudinal mixed-method design. The study was advertised to community programs (e.g. Area Agencies on Aging, Alzheimer’s Association), and N = 83 caregivers completed the post-intervention survey (Mean age = 62.6; 82% female; 89% White, 57% spouse caregivers). The system usability scale averaged 86.1/100, indicating high acceptability, with the majority of participants reporting they would like to use the program again (76%), that the information was helpful (86%), and that they would refer the program to other caregivers (85%). We used a team-based approach to thematic analysis of in-depth interviews from 28 participants at both post-intervention and follow-up. Qualitative data supported the quantitative findings as participants expressed a high level of satisfaction with the program’s online asynchronous structure and content. Participants emphasized the practical skill-building exercises and emotional education as particularly helpful. Further, many participants reported continued engagement with the program by revisiting notes, reviewing session summaries, and reflecting on specific analogies or information. Those who maintained engagement away from the program reported increased motivation to apply skills taught in the program in their daily lives. The online self-guided ACT for Caregivers program demonstrates the potential for broader implementation of ACT in community and healthcare settings through its mobile and cost-effective nature.

